# Getting to the First 90: Incentivized Peer Mobilizers Promote HIV Testing Services to Men Who Have Sex With Men Using Social Media in Mumbai, India

**DOI:** 10.9745/GHSP-D-19-00094

**Published:** 2019-09-23

**Authors:** Anjana Das, Bitra George, Virupax Ranebennur, M. R. Parthasarathy, G. S. Shreenivas, Priyamvada Todankar, Amit Shrivastav, Ajay Kumar Reddy, Christopher Akolo, Michael Cassell, Sandeep Mane, Deepak Tripathi, Jiban Baishya

**Affiliations:** a FHI 360, New Delhi, India. Now an independent consultant.; b FHI 360, New Delhi, India.; c FHI 360, Washington, DC, USA.; d FHI 360, Bangkok, Thailand.; e Humsafar Trust, Mumbai, India.; f United States Agency for International Development, New Delhi, India.

## Abstract

Recent studies of Indian men who have sex with men (MSM) have shown widespread use of social media for seeking sex partners. We piloted a peer mobilization approach to explore the feasibility of engaging previously unreached MSM online to link them to HIV testing services (HTS). MSM were encouraged to seek HTS through messages posted on a popular dating website. Those who visited the designated HTS site and tested for HIV were recruited as peer mobilizers and given coupons with unique identifying codes to distribute to other men in their virtual networks. If a network member presented at the site with a coupon and tested for HIV, the peer mobilizer was given a small monetary incentive. Network members presenting at the testing site were also recruited as peer mobilizers and given coupons. In a 6-month period, 247 MSM were recruited and tested for HIV and syphilis, of whom 244 (99%) were first-time testers. Two-thirds were less than 25 years old and about half reported inconsistent or no condom use during the last 10 anal sex acts. Eight individuals (3.2%) tested positive for HIV, and 22 (8.9%) had a high titer for syphilis; all were referred to tertiary hospitals for treatment. Our approach was modestly successful in reaching and providing HTS to previously unreached MSM, but challenges included lower-than-expected recruitment, individuals not returning for posttest counseling, and loss to follow-up of individuals with HIV. The next phase of peer mobilization will aim to scale up these services through government-supported targeted interventions for this subpopulation of primarily young, unreached MSM at high risk. The challenges will be addressed by targeting more dating sites, increasing access to testing using rapid HIV tests at several community-based facilities, and offering peer navigation support for people living with HIV.

## Abstract (precis)

This peer mobilization pilot for HIV and syphilis testing used messaging on gay dating sites, clinic referrals, and peer recruitment to reach men who have sex with men in Mumbai. In 6 months, the pilot reached a relatively modest 247 individuals, 244 of whom had never tested for HIV. Challenges included low recruitment and loss to follow-up for posttest counseling and treatment initiation for individuals with HIV.

## INTRODUCTION

The Joint United Nations Programme on HIV/AIDS (UNAIDS) has set ambitious 90-90-90 targets to accelerate the end of the HIV epidemic—that is, by 2020, 90% of people living with HIV (PLHIV) will know their HIV status, 90% of people diagnosed with HIV infection will receive sustained antiretroviral therapy (ART), and 90% of people receiving ART will have viral suppression.^1^ Despite significantly increased access to ART among PLHIV, reduction in the rates of new HIV infections has been less substantial (16%) between 2010 and 2016.^2^ Various factors explain this lack of progress, including the possibility that an estimated 30% of PLHIV remain undiagnosed and untreated and thus continue to transmit the virus to uninfected sexual or injecting partners. Better testing approaches are needed to reach populations for which HIV risk is highest and HIV prevention, testing, and treatment coverage is lowest in order to achieve the first 90 target.^3^

India has an estimated 2.1 million PLHIV and a concentrated epidemic.^4^ The National Integrated Biological and Behavioral Surveillance (IBBS) conducted in 2014–2015 among men who have sex with men (MSM) using cluster sampling showed a national HIV prevalence of 4.3% and 4.9% in the state of Maharashtra.^5^ The 2016–2017 round of HIV Sentinel Surveillance conducted among a random sample of MSM registered with targeted interventions showed an all-India prevalence of 2.69% and a prevalence of 4.69% in Maharashtra.^6^ Analysis of national-level surveys of Indian MSM showed that significant factors associated with HIV positivity were being the receptive partner or both receptive and penetrative as compared to exclusive penetrative partners; being more than 25 years old as compared to younger counterparts; being illiterate rather than literate; and being employed versus unemployed.^7^ An HIV cascade study in MSM from 12 cities using respondent-driven sampling showed an HIV prevalence of 9.5%, but 70% of PLHIV were not aware of their status because they had tested more than a year earlier or had never tested.^8^ The national program supports targeted interventions for key populations including MSM; these interventions are implemented by NGOs and provide prevention services and commodities, referrals to integrated counseling and testing centers (ICTC) for HIV and syphilis testing, and referrals to ART centers for PLHIV. Targeted interventions provide services to MSM who are physically present at “hot spots”—sites where soliciting and/or sexual activities take place, such as public toilets. However, several studies in India show that many MSM use social media and other web-based platforms to seek sex partners.^9^^–^^11^ The movement from conventional physical locations to virtual spaces poses a challenge in reaching this “hidden” subpopulation of MSM.

Many MSM in India use web-based platforms to seek sex partners, creating a hidden subpopulation that may be difficult to reach.

The Linkages across the Continuum of HIV Services for Key Populations Affected by HIV (LINKAGES) Project in India works with government-supported targeted interventions to promote the HIV continuum of care for key populations in 6 districts with high HIV prevalence in 2 states, including the large metropolitan city Mumbai in the state of Maharashtra. A LINKAGES baseline survey (October 2015 to March 2016) in Mumbai showed that 8,684 MSM were registered and receiving regular services from targeted interventions, and 209 of these men were PLHIV (2.4% prevalence). Community discussions revealed a hidden population that remain unreached through the traditional hot spot–based outreach program of targeted interventions. The reasons included an increasing trend of seeking sex partners on social media/mobile phones instead of hot spots and a reluctance to avail targeted interventions for fear of disclosure of status and/or identity leading to social stigma and criminalization. We piloted a peer mobilization approach to explore the feasibility of connecting with unreached virtual networks of MSM in Mumbai who may not visit hot spots and promoting HTS within these networks. This article describes the implementation of the approach and lessons learned during a 6-month period from January to July 2017.

We piloted a peer mobilization approach for connecting with unreached virtual networks of MSM in Mumbai and promoting HTS within these networks.

## METHODS

The network-based peer mobilization approach is inspired by the successful use of respondent-driven sampling, a method used to reach a community-based sample of hidden or hard-to-reach populations for HIV surveillance.^12^ During implementation of the peer mobilization approach, MSM were contacted through messages posted on social media; respondents who made use of HTS at the designated site were, if willing, recruited as “primary seeds” or first-wave peer mobilizers. Peer mobilizers were oriented on motivating peers for HTS and provided 4 coupons each with unique identifying codes. During physical interactions, peer mobilizers gave the coupons to peers who used social media for soliciting partners and were interested in taking up the HIV services on offer. If a peer visited the designated site with the coupon, consented to HIV testing, and underwent testing, the peer mobilizer was given a small monetary incentive of 300 Indian Rupees (INR) (US$5) for his effort. Participants who attended the HTS site were given a travel reimbursement of 150 INR (US$2.50). Referred peers, if willing, were also enrolled as peer mobilizers and provided a similar number of coupons. In this fashion, several waves of peer mobilizers were recruited in each network generated by a primary seed.

### Preparatory Phase

We named the peer mobilization project *Mulakat*, a Hindustani word that means “meeting.” The designated testing site for the project was Humsafar Trust (HST), an MSM community-based organization office/drop-in center, clinic, and ICTC located in suburban Mumbai. HST's prior experience with online surveys had shown that the most commonly used websites of Mumbai MSM were PlanetRomeo, Facebook, and Grindr.^13^ We decided to target PlanetRomeo because HST had an ongoing agreement with the website managers for posting messages free of cost. A community consultation was organized to develop messages about Project *Mulakat* to be posted on the site. The messages pertained to community members' roles in maintaining a safe MSM community and the benefits of availing HTS at HST.

The LINKAGES team designed and printed coupons with unique code numbers, validity periods, and contact details of HST, as well as a bespoke tool to record participants' sociodemographic profile and risk behaviors. A coupon manager based at HST was the point of contact for MSM attending the clinic. He ensured MSM fulfilling eligibility criteria received all services (pre- and posttest counseling, blood tests, clinical check-up, referrals for treatment of individuals with positive results for HIV/syphilis), enrolled peer mobilizers, tracked coupons, and maintained individual records, which were summarized and reported monthly. In addition, 3 Internet outreach workers from the MSM community created their own profiles and posted messages on PlanetRomeo in defined geographical areas and directed respondents to the coupon manager. Internet outreach workers were hired for a period of 3 months; each was given a target of recruiting 7 primary seeds and, in coordination with the coupon manager, followed up with peer mobilizers for coupon disbursals to peers. The HST and LINKAGES management teams monitored activities and results at frequent intervals.

### Inclusion Criteria and Client Flow

**FIGURE 1:**
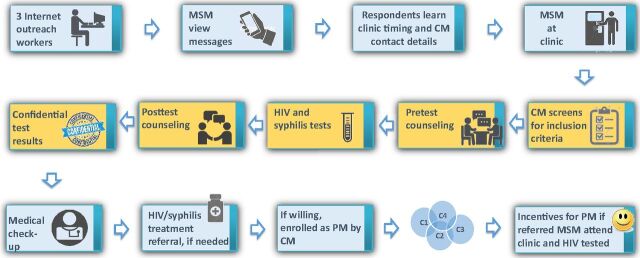
Client Flow Abbreviations: C, coupons given to PM; CM, coupon manager; MSM, men who have sex with men; PM, peer mobilizer.

The client flow is shown in Figure 1. MSM attending the HST clinic who did not meet eligibility criteria were provided services as per their requirements but not enrolled in the project (Box 1). Eligible MSM were asked for information pertaining to sociodemographic profile and risk behaviors and offered pretest counseling. Those who gave written informed consent (as per norms followed by the HST ICTC) were tested for HIV and syphilis and asked to return the next day for posttest counseling. Individuals with positive test results for HIV, syphilis, or both were referred to tertiary hospitals. All participants received prevention education during pre- and posttest counseling, were offered assistance for registering with targeted interventions for ongoing services, and received prevention messages from the coupon manager at regular intervals via WhatsApp in which group members could not view others' contact details. At the posttest visit, MSM willing to be enrolled as peer mobilizers were oriented to the project by the coupon manager and given tips on how to motivate other MSM to avail HTS. Coupons given to peer mobilizers had a validity period of 30 days. If none or only some of the coupons had been used within the time period, peer mobilizers were contacted and requested to encourage their peers to attend the clinic.

BOX 1 **Eligibility Criteria for Enrollment of MSM in Project *Mulakat***

- At least 18 years of age
- Accessed social media to seek male sex partners in the last 3 months
- Had sex with a male in the previous month
- Not availing services from or registered with existing government-supported targeted intervention programs for MSM

### Laboratory Tests

Syphilis testing was done using the rapid plasma reagin kit manufactured by Span Diagnostics. A titer of 1:8 or more was considered as high-titer syphilis, and individuals with such results were referred to a tertiary hospital for further evaluation and treatment as per national guidelines. For HIV testing, the ICTC at HST followed the national guidelines of 3 tests for asymptomatic individuals using different kits in a particular order, with the subsequent test being performed only if the previous test result was positive. The first test kit used was COMBAIDS, followed by MERISCREEN and thereafter AIDSCAN.

The national program approved and issued guidelines for HIV screening at targeted intervention sites using rapid tests in December 2016.^14^ Rapid testing was operationalized after Project *Mulakat* was completed.

### Data

The results were derived from secondary analysis of routine service statistics. The protocol for analysis was reviewed by FHI 360's Protection of Human Subjects Committee and given a nonresearch determination.

## RESULTS

**FIGURE 2:**
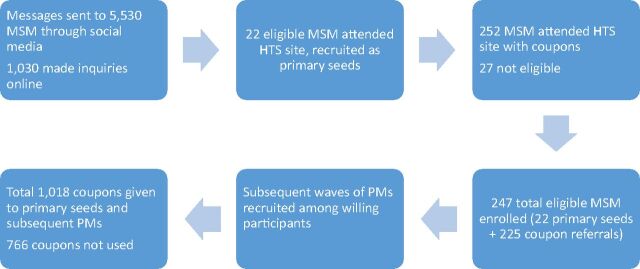
Peer Mobilization: Process and Output Indicators Abbreviations: HTS: HIV testing services; MSM, men who have sex with men; PM, peer mobilizer.

Process data and outputs of the peer mobilization intervention are shown in Figure 2. In the period January to July 2017, messages on social media were sent to 5,530 MSM and 1,030 MSM made online inquiries. Through social media and coupon referrals, a total of 274 individuals attended the clinic, of whom 27 were ineligible because they either had received targeted intervention services (n=23) or were less than 18 years old (n=4). Thus, 247 MSM were enrolled, which included 22 primary seeds (first-wave peer mobilizers), subsequent waves of peer mobilizers, and others unwilling to be peer mobilizers. The numbers of MSM recruited from each network generated from the 22 primary seeds (not shown) varied greatly. The mean size of the 5 largest networks was 39.8 (range 13–81), while the mean size of 11 networks was 3.8 (range 1–7); 6 primary seeds did not refer others.

A profile of the participants is shown in the Table. Two-thirds (69%) were less than 25 years old and their preferred social media platforms were Facebook, Grindr, IMO, Instagram, PlanetRomeo, Tinder, and WhatsApp. Nearly half (44%) reported inconsistent or no condom use during the last 10 acts of anal sex, and some reported other high-risk behaviors such as transactional sex, group sex, and substance use during sex. Among the 247 participants, 244 (99%) were first-time testers. The prevalence of HIV and high-titer syphilis was 3.2% and 8.9%, respectively. Half of those with HIV diagnosed were successfully linked to treatment, and all but one of those with a positive test result for syphilis attended the referral hospital for treatment.

**TABLE.** Participants' Sociodemographic Profile and Risk Behaviors and HIV and Syphilis Prevalence (N=247)

Characteristics	No. (%)
**Age group, years**	
<20	70 (28)
20–24	101 (41)
>25	76 (31)
**Occupation**	
Student	111 (45)
Service	63 (26)
Others	73 (29)
**Faced violence during/after sex with male partners**	
Yes	25 (10)
No	222 (90)
**Preferred social media platforms**	
Grindr	161 (22)
WhatsApp	143 (20)
Facebook	139 (19)
PlanetRomeo	65 (9)
Others	218 (30)
**Received cash or kind for sex with a man in previous year**	
Yes	59 (24)
No	188 (76)
**Paid cash or kind for sex with a man in previous year**	
Yes	22(9)
No	225 (91)
**Participated in group sex**	
Yes	27 (11)
No	220 (89)
**Uses alcohol and/or drugs during sex**	
Sometimes/often	73 (30)
Never	174 (70)
**Condom use during anal sex (last 10 acts)**	
Consistent	139 (56)
Inconsistent	87 (35)
Never	21 (9)
**Never tested for HIV previously**	244 (99)
**Positive HIV test result**	8 (3.2)
**High-titer syphilis (RPR titer ≥ 1:8)**	22 (8.9)

Abbreviation: RPR, rapid plasma reagin.

### Achievements

#### Reached Previously Unreached MSM at Risk of HIV

The peer mobilization approach was able to reach and provide HTS to MSM who had never tested for HIV and were outside the ambit of government-supported targeted intervention services. The poor testing rates were in contrast to the IBBS in which 88.2% of Maharashtra MSM reported prior testing for HIV. We probably reached a different subgroup by operating online, in contrast to the IBBS, which had recruited people from physical hotspots. Our subpopulation of MSM had a lower HIV prevalence (3.2%) as compared to that in IBBS Maharashtra (4.9%), which could be due to the MSM in our study being younger—69% were under 25 years as compared to 34.5% in the Maharashtra IBBS—and thus having fewer years of risk behavior. Also, as mentioned earlier, Indian MSM studies showed that being more than 25 years of age is a significant factor associated with an HIV-positive status. However, the HIV prevalence in our MSM sample was higher than among those registered with targeted interventions (2.4%) despite the latter being an older group (only 22.2% were less than 25 years old). MSM reached through the peer mobilization approach appeared to be at high risk of HIV because of unprotected anal sex (44%) and had a high prevalence of syphilis (8.9%). The program was well received by the community; some peer mobilizers donated their incentives to the HST PLHIV support group, saying their only motive in getting enrolled as peer mobilizers was to help other community members.

The peer mobilization approach was able to reach and provide HTS to MSM who had never tested for HIV.

#### Provided Referrals for MSM With HIV or Syphilis

Project *Mulakat* used the existing HST referral systems to tertiary care hospitals. All high-titer syphilis cases were referred to a particular government hospital where an HST staff member was posted; he ensured that these individuals received further management. Of a total of 22 persons with high-titer syphilis, 21 attended the referral hospital while 1 did not attend, citing inconvenient timings. Individuals with HIV were referred to different ART centers closest to their residence as per government norms, and they were also offered support through the HST PLHIV network. Of the 8 MSM receiving an HIV-positive diagnosis, 4 were initiated on treatment.

#### Kept Costs Low by Using Existing Target Intervention Services

The direct cost of the intervention for the 6-month period was 260,000 INR (US$4,333), which included personnel, material, and incentive costs. Indirect costs such as clinic and laboratory staff time and supplies were borne by HST through the targeted intervention and ICTC budget.

### Challenges

#### Lower-Than-Expected Recruitment

**FIGURE 3:**
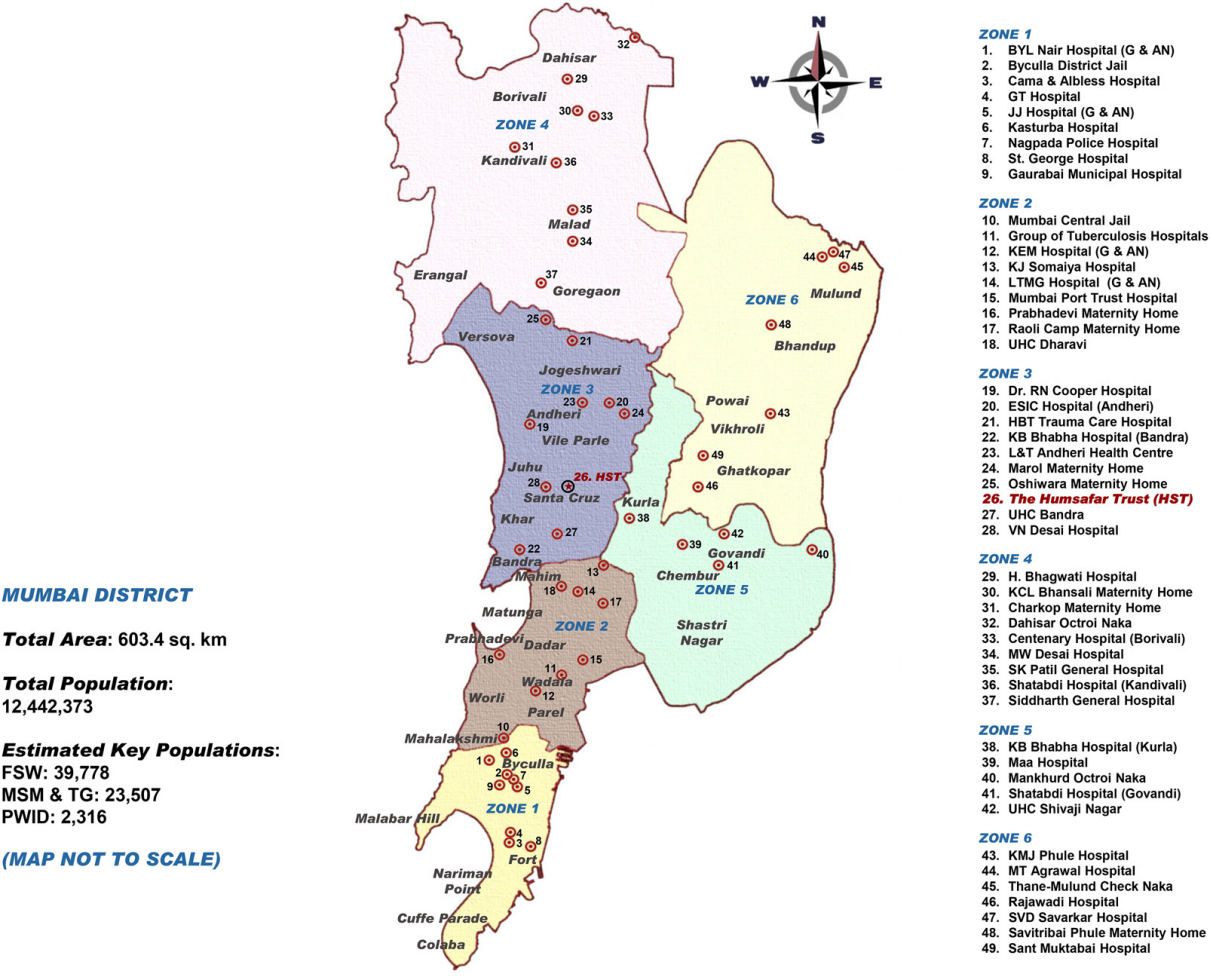
Government-Supported HIV Testing Services in Mumbai, India Abbreviations: FSW, female sex worker; MSM & TG, men who have sex with men and transgender women; PWID, persons who inject drugs; G, General HIV testing services for all; AN: Exclusive HIV testing services for antenatal women. Source of total population data: Population Census 2011. Mumbai (Greater Mumbai) City Census 2011 Data. https://www.census2011.co.in/census/city/365-mumbai.html. Accessed July 6, 2019. Source of key population data: India Health Action Trust. *HIV/AIDS Situation and Response in Mumbai City and Suburban Districts: Epidemiological Appraisal Using Data Triangulation*. Bangalore, India: India Health Action Trust; 2010.

Project *Mulakat* was based on the LINKAGES Thailand program in which incentivized peer mobilizers recruited 424 network members over a 5-month period. However, the Thailand methodology differed in that peer mobilizers recruited both online and offline contacts. In addition, the peer mobilizers passed on contact information of willing peers to trained, salaried outreach workers (called community-based supporters) who contacted them directly for HTS.^15^ The possible reasons for lower numbers in Project *Mulakat* include the following:

- **Limited websites:** We initially posted messages on PlanetRomeo due to an existing service agreement between HST and PlanetRomeo and added Facebook at a later stage. However, participants stated a preference for other social media platforms, such as Grindr and WhatsApp.
- **Low utilization of coupons:** Many coupons issued to peer mobilizers did not convert into clinic visits by peers; either the peer mobilizer did not distribute the coupons or the persons given the coupons did not go to the facility. A factor that may have affected coupon utilization was the use of a single testing site. Mumbai is a large city, and traveling to the HST site may not have been convenient for those living far away. Figure 3 is an administrative zone map of government-supported Mumbai ICTCs, with HST in zone 3. In addition, Mumbai has 4 mobile ICTC vans and 24 targeted intervention sites capable of providing community-based HIV screening that could also be utilized during the scale-up phase.
- **Small networks:** Another likely factor in the low recruitment was that most peer mobilizers were members of small networks. Of a total of 22 networks, only 5 had more than 10 members (range 13–81) who attended the HST clinic. Several MSM were unwilling to be recruited as peer mobilizers, and one of their main reasons was that they did not have enough contacts in the community. The Thailand program also reported that only 20% of participants agreed to take on peer mobilizer roles, but a single super-recruiter successfully reached 149 new clients in 6 months, of whom 93% received an HIV test. The next phase of peer mobilization in India should, through community consultations, attempt to identify peers with large networks prior to implementation.
- **Stigma and discrimination:** Homosexuality was decriminalized in India in September 2018, which was after the project ended. Participants were offered facilitated registration at targeted interventions for ongoing services, but very few accepted the offer. Fear of law, stigma, and discrimination may have prevented some individuals from participating in the project.
  Fear of law, stigma, and discrimination may have prevented some individuals from participating in the project.

Many coupons issued to peer mobilizers did not convert into clinic visits by peers, which contributed to lower-than-expected recruitment.

#### Individuals Not Returning for HIV Test Results

During the project period, 50 MSM did not return to the clinic for posttest counseling despite repeated reminders; among them, 1 had tested positive for HIV. Of the 50 men, 40 collected their test results after the project period. The most common reason given for the delayed visit was that they were students in Mumbai and had gone to their hometown for holidays. The challenge can be addressed by using rapid tests for HIV screening with same-day test results, which are now available at targeted interventions.

#### PLHIV Lost to Follow-Up

Among the 8 MSM who tested positive, 4 were registered with ART centers and treatment was initiated. Of the remaining PLHIV, 1 did not return to collect test results, 2 refused to accept their positive status in spite of several counseling sessions, and 1 relocated to his hometown. A review of social media strategies for promoting HIV service uptake along the continuum of care within key populations showed that interventions around linkages to and retention in care and initiation of ART need further development.^16^ Peer navigation for facilitating referrals to government-run ART centers and social support by MSM PLHIV networks could help promote treatment linkages and adherence support for PLHIV.

## LESSONS LEARNED

Several factors contributed to the achievements of the peer mobilization pilot in Mumbai:

- **Detailed planning with community involvement:** The planning exercise for Project *Mulakat* took about 2 months and was led by LINKAGES staff with support from HST staff from other projects and community leaders. Operational guidelines were developed for the entire process with timelines, client flow, clinic and monitoring formats, and an indicative budget.
- **Message development:** Involvement of the community in message development ensured that messages posted on websites were innovative and caught the attention of the virtual MSM community. Some of the online messages are shown in Box 2.
- **Data-driven activity modifications:** Regular monitoring of project outputs by the LINKAGES and HST management teams resulted in some modifications to activities during the course of the project. For example, when recruitment of primary seeds took longer than anticipated, we decided to post messages on Facebook in addition to PlanetRomeo. To address the challenge of coupons not being utilized, 2 community events were organized at the HST drop-in center. Peer mobilizers were informed and asked to encourage their peers to attend the events and present coupons for availing services. Through this initiative, 21 people were reached and tested. In addition, at a later stage, peer mobilizers who said they had a large network were given more than 4 coupons on request.

BOX 2 **Messages Posted Online**

- HIV tests make me feel sexy and safe. When was the last time you tested for HIV? To know how to get a test for you and your friends, talk to us.
- Don't lose this opportunity to be a star among your friends. To educate your friends about HIV, join us today!
- Let our confidential services build your trust toward taking a step to being safe from HIV! To be a part of our mission, please call …

Specific measures that can be taken during scale-up to address the challenges encountered in Project *Mulakat* are provided in Box 3.

BOX 3 **Recommendations for Scaling Up Peer Mobilization Activities Through Targeted Intervention**

- Use trained and salaried outreach workers dedicated for peer mobilization activities
- Partner with multiple popular dating sites to gain access to a large user base
- Tap multiple sites for HIV testing services using rapid tests, including existing integrated counseling and testing centers, mobile vans, and community-based screening by targeted interventions
- Use assisted self-testing at a later stage once kits are available locally
- Focus on identifying and tracking large, high-risk networks in which a member is positive for HIV/syphilis
- Promote treatment initiation and adherence through the use of accompanied referrals to ART centers, peer navigation/people living with HIV network support, and referrals to community-based ART centers where available

## CONCLUSIONS AND NEXT STEPS

Project *Mulakat* demonstrated the feasibility of connecting with unreached virtual networks of urban Indian MSM to promote HTS and generated practical recommendations for improving the effectiveness of the intervention. To provide follow-up services, global experiences of innovative programs using communication technology can be used to develop a combination package of online-to-offline interventions for MSM.^17^^–^^19^ These interventions may include online sessions on risk assessment and reduction and clinic visits for HTS and tests for sexually transmitted infections.

The strategic approach for MSM interventions under the National AIDS Control Organization (NACO) provides guidelines to targeted interventions for additional technology-based outreach including use of MSM dating websites.^20^ The peer mobilization approach can be integrated into targeted intervetion programs for reaching MSM not found at hot spots but active on social media. In the next phase, LINKAGES, in collaboration with NACO, will develop an intervention package for virtual subpopulations of MSM that can be implemented at a greater scale by targeted interventions. The lessons learned from Project *Mulakat*, as well as other experiences with MSM networks using social media in different regions of India and peri-urban/rural settings, will inform the intervention package and help refine national guidelines to reach and provide services to this hard-to-reach subset of the MSM population.

## Notes

**Peer Reviewed**

**Funding:** This publication is made possible by the support of the American People through the United States Agency for International Development (USAID) and the United States President's Emergency Plan for AIDS Relief (PEPFAR) through the LINKAGES project, cooperative agreement number AID-OAA-A-14-00045.
